# Indigenous Food Systems Changes and Resiliency: Protocol for a Scoping Review

**DOI:** 10.2196/41627

**Published:** 2023-04-21

**Authors:** Hiliary Monteith, Elizabeth Claire Hiscock, Yasamin Sadeghi, Emily V Smith, Angela Mashford-Pringle

**Affiliations:** 1 Department of Nutritional Sciences University of Toronto Toronto, ON Canada; 2 Rehabilitation Sciences Institute University of Toronto Toronto, ON Canada; 3 Dalla Lana School of Public Health University of Toronto Toronto, ON Canada; 4 Department of Anthropology University of Toronto Toronto, ON Canada; 5 Waakebiness-Bryce Institute for Indigenous Health University of Toronto Toronto, ON Canada

**Keywords:** Indigenous, food systems, Indigenous health, scoping review, traditional foods, colonization, climate change, pollution, environment

## Abstract

**Background:**

Indigenous food systems (IFS) consider the complex relationships and connections between land, animals, plants, water, and people. These food systems may differ between regions, Indigenous cultures, and history; however, given the similar colonial histories and policies influencing Indigenous groups in Canada, the United States, Australia, and Aotearoa (New Zealand), the IFS changes and responses in these regions may follow similar trends. Climate change and pollution continue to impact the environment in catastrophic ways, and this, in turn, impacts IFS. However, to date, there has been no review of the literature on IFS, how they are changing, and how communities are responding to these changes.

**Objective:**

In this scoping review, we will summarize primary research in Canada, the United States, Australia, and Aotearoa related to IFS addressing the following questions: (1) What changes are IFS experiencing in the context of climate change and pollution? (2) What actions have been taken in response to IFS changes? (3) What are the characteristics of IFS research in peer-reviewed academic literature?

**Methods:**

We will use the PRISMA (Preferred Reporting Items for Systematic Reviews and Meta-Analyses) guidelines for scoping reviews and the Joanna Briggs Institute reviewer’s manual to inform the review process. MEDLINE, SCOPUS, International Bibliography of the Social Sciences, Sociological Abstracts, and the Bibliography of Native North Americans are the databases included in this review search. All screening and extraction have been supported by Covidence software (Veritas Health Innovation) with 2 independent reviewers conducting the abstract and full-text screening. We will map concepts and themes related to the research questions to contribute to the understanding of IFS within the academic literature and provide a narrative review of the outcomes.

**Results:**

The electronic database searches for this review were conducted in May 2021. Screening and full-text review were initially completed in the winter of 2022. We are currently in the process of compiling results and aim to share findings in 2023.

**Conclusions:**

This review will provide valuable insight into current IFS needs by summarizing the peer-reviewed literature on how IFS are changing because of climate change and pollution and how communities are responding to these changes. The results of this review will be shared with Indigenous communities, through academic publications, community conversations, and conference presentations.

**Trial Registration:**

OSF Registries osf.io/xrj87; https://osf.io/xrj87

**International Registered Report Identifier (IRRID):**

RR1-10.2196/41627

## Introduction

Indigenous food systems (IFS) refer to the connections between Indigenous groups and the land; however, climate change, colonization, and pollution have resulted in detrimental impacts to the environment and access to it [1]. This influences food sovereignty and security for Indigenous groups in Canada, the United States, Aotearoa (New Zealand), and Australia in similar ways due to the shared impacts of Western-European colonization [2,3]. Food sovereignty refers to the sovereign rights of Indigenous Peoples to self-determine their own food systems, connecting with land, history, language, and ways of life [4]. Disruptions to food sovereignty can result in food insecurity, an issue disproportionately impacting Indigenous communities with prevalence rates consistently above 25% in Canada, the United States, Australia, and Aotearoa [5-7]. Additionally, inconsistent definitions and measures of food insecurity between regions and institutions may lead to underestimations of food insecurity among Indigenous communities [8].

Household food insecurity has several health implications including a greater prevalence of diabetes, heart disease, asthma, musculoskeletal diseases, intestinal disorders, and mental health disorders [9,10]. Food insecurity is more prevalent in households with children [5], and therefore it is important to consider health concerns throughout the lifespan. For example, children and adolescents in homes experiencing food insecurity are more likely to have suicidal ideation, mood disorders, and asthma [10,11]. Although much of the research to date has focused on physical and mental health implications, food insecurity is a complex public health concern that is deeply intertwined with Indigenous spiritual and emotional health and overall well-being [12].

To date, there have been no systematic explorations of IFS changes in the peer-reviewed literature. Therefore, this scoping review will characterize recent trends in peer-reviewed research concerning IFS changes and Indigenous food sovereignty with the objective of understanding the experiences of Indigenous Peoples living in Canada, the United States, Australia, and Aotearoa and how communities have observed and responded to changes in their traditional lifeways. IFS changes refer to broad adaptations to market and land-based food systems including both positive and negative impacts. For example, an IFS change could be the reduction of an animal population or a response to the loss of hunting skills where a community is educating youth through on-the-land hunting activities. This review will discuss the potential impacts of climate change and pollution on IFS, provide information on the significance of IFS, and explore how IFS have been changing in similar and different ways in these regions. Although each food system lies within a unique context, there is considerable similarity in the experiences of Indigenous Peoples within these regions, particularly as it relates to colonization [2,3]. This review will help identify those points of similarity and difference to generate a better understanding of the issues in IFS and facilitate connections between Indigenous communities and their shared experiences. The following research questions are of interest: (1) What changes are IFS experiencing in the context of climate change and pollution? (2) What actions have been taken in response to these IFS changes? and (3) What are the characteristics of IFS research in peer-reviewed academic literature?

## Methods

### Ontological Origins

This scoping review emerged from discussions between Indigenous scholars, academics, Elders, and community members who were focused on IFS as part of a proposed partnership between the University of Toronto, Canada and Moi University, Kenya. The creation of this review is tied to the goals of this partnership and informed by initial feedback from the community of practice in 2020 and 2021. In accordance with Indigenous communal decision-making traditions in Canada, all decisions were open to input from members of the community of practice with the goal of collective consensus on the direction of the project. Solicitation of feedback was facilitated by regular group meetings and the dissemination of project updates via email by those who were unable to attend meetings.

### Eligibility Criteria

The eligibility criteria (Textbox 1) were amended following initial screening and calibration due to the significant number of citations involved. To maintain a focused scope, we excluded work that focused on colonization as a driver of environmental change and only included papers that primarily described climate change and pollution as influencing environmental changes. Works that only discussed environmental revitalization were also excluded as these papers differed significantly from the others and may require a separate review. We retained papers specific to colonization and revitalization so that we could do an additional review and synthesis if desired at a later date, as we recognize the value in these topics.

**Eligibility criteria.**

**Inclusion criteria**

- Directly related to Indigenous Peoples living in Canada, the United States, Australia, or Aotearoa
- Related to Indigenous food systems or Indigenous food sovereignty
- About Indigenous food systems changes or actions to protect or revitalize Indigenous food systems related to climate change or pollution
- Primary research
- Published between 2016 and 2021
- Written in English
- Available electronically

**Exclusion criteria**

- Not related to Indigenous Peoples or Indigenous Peoples specifically residing in Canada, the United States, Australia, or Aotearoa
- Not related to Indigenous food systems or food sovereignty
- Not related to Indigenous food systems changes or actions to protect or revitalize Indigenous food systems related to climate change or pollution
- Not primary research (does not include data collection)
- Not published between 2016 and 2021
- Not available in English
- Papers that only document Indigenous foods (ethnobotany)
- Papers that only document the food consumption of Indigenous Peoples
- Papers that only document food literacy of Indigenous People
- Papers about teaching Indigenous Peoples how to grow or cook non-Indigenous foods or use capitalist market-based retail environments
- Papers focused on food security or insecurity for Indigenous Peoples without discussing Indigenous food systems and experiences or changes related to climate change or pollution
- Papers about Indigenous environmental management unless it explicitly includes information about Indigenous food systems

### Information Sources

This protocol was developed using the methodological framework for scoping reviews outlined in the PRISMA-ScR (Preferred Reporting Items for Systematic Reviews and Meta-Analyses Extension for Scoping Reviews) guidelines [13] and informed by the Joanna Briggs Institute Reviewers’ Manual [14]. An electronic search was conducted in the following databases: MEDLINE, SCOPUS, International Bibliography of the Social Sciences, Sociological Abstracts, and the Bibliography of Native North Americans for academic literature relating to IFS across the globe. The search strategy was developed to find journal articles focused on IFS changes or the protection or revitalization of IFS, and with the knowledge that the language used to describe those ideas can vary between countries and disciplines. Index terms were used for each database where index terms were available. Additionally, some databases may use terms that are no longer in use to refer to Indigenous Peoples, which is a limitation of the search functions. We took great care to find the articles within the limits of the databases despite knowledge of the outdated terms. All database searches were conducted on June 1, 2021. Citations obtained from the searches were exported to Covidence for the removal of duplicates and used for screening and data extraction [15]. Reference lists will not be searched for additional citations due to capacity limits. Documentation of the search strategy can be found in Multimedia Appendix 1.

### Selection of Sources of Evidence

Two independent reviewers completed both the abstract and full-text screening using Covidence software [15] to facilitate the process, aligning with PRISMA (Preferred Reporting Items for Systematic Reviews and Meta-Analyses) guidelines. A sample of 10 papers was assessed by authors ECH and EVS to test the validity of the eligibility criteria, after which the 2 reviewers compared the screening results to calibrate the criteria and ensure a high level of rater interreliability. After calibration, screening proceeded until complete. The full-text review was completed by authors HM, ECH, YS, and EVS. All conflicts were resolved by meeting among the reviewers and reaching a consensus. Disagreements were solved by consensus, and if consensus could not be reached, the disagreements were resolved by a third reviewer.

### Data Charting Process

Data extraction will be completed using a detailed template, and pilot testing will be completed with the team prior to moving forward with all data extraction. Once the data extraction tool has been adjusted, the data extraction will proceed independently. Should any clarifications be required throughout the process, the team will meet to ensure consistency with extraction. Textbox 2 provides detail on the data extracted for this review.

**Data extraction tool details (the data extraction tool collected information from 4 main categories with specific information represented below them).**

**Paper information**

- Title
- First author
- Publication year
- Positionality of authors or researchers
- Additional notes

**Location**

- Country
- Specific Location details (ie, province)
- Type of region (ie, remote)
- Type of climate (ie, Boreal Forest)
- Additional notes

**Study details**

- Aim and objective
- Study design
- Dates and time
- Funding sources
- Indigenous community or organization
- Indigenous group/People
- Name of the nonhuman population (ie, Geese)
- Number of participants
- Indigenous engagement (yes/no and explain)
- Additional notes

**Results**

- Food system category (ie, food acquisition)
- Food system disruptors (ie, pollution)
- Food system changes
- Are food system changes the primary focus? Elaborate
- Policy recommendations
- Knowledge dissemination and translation
- Impact on community

### Data Items

We are interested in collecting information on how the studies were conducted, where the study was done and with or for whom, the details describing the IFS, the changes related to the environment (eg, climate), and the community perspective and response to the food systems changes. The data extraction template addresses each of these key topics to enable us to answer the research questions of interest. Studies that only discuss changes related to colonization and community responses without describing specific IFS changes will not be captured in this review.

For this review, “Indigenous Peoples” are defined as those who self-identify as Indigenous. In Canada, Indigenous groups may include Inuit, Métis, and First Nations, including any of the over 600 First Nations communities. In Aotearoa, the Māori people are the Indigenous Peoples of the land, in the United States, Native American Peoples, American Indians, and Alaska Natives, and in Australia, this includes Aboriginal and Torres Strait Islanders. We recognize that not all Indigenous groups identify with the same terminologies, and so we aim to be inclusive and respectful of local terminology; however, previous publications included in this review may use outdated terms. We will aim to use the most up-to-date terminology even if it differs from language used in previous publications.

“Indigenous food systems” (IFS) are any traditional foods and foodways that any self-identified Indigenous group living on the lands known today as Canada, the United States, Aotearoa, and Australia defines as such. Generally, these systems are deeply connected to the lands and environments from which they emerged within these regions.

### Synthesis of Results

Results will be presented both quantitatively and qualitatively by providing numerical and descriptive details of the papers included. We will map the findings and conceptualizations of this work, and present the number of studies screened, included, and excluded at each stage of this review using tables and diagrams. The results will be mapped into categories based on research objectives (ie, climate change) and themes will be described. In addition to mapping and describing the outcomes specific to environmental changes and community responses to these changes, we will compare the methodologies used and discuss these approaches in detail. In addition, it is important to note that we will include Indigenous community and scholar involvement in the interpretation and mapping of this work.

### Ethical Considerations

All data in this review will be gathered through database searches of primary research and does not include identifiable individual data. Only secondary analyses will be conducted; therefore, no research ethics approval is required for this scoping review. This protocol was registered with the Open Science Framework on January 13, 2022 (10.17605/OSF.IO/XRJ87).

## Results

The electronic database searches for this review were conducted in May 2021. Screening and full-text review were initially completed in the winter of 2022. We are currently in the process of compiling results and aim to share findings in 2023.

## Discussion

We describe the protocol for a scoping review of academic, peer-reviewed literature about IFS changes and Indigenous food sovereignty in Canada, the United States, Australia, and Aotearoa. To our knowledge, no review of the literature on IFS changes and community responses related to climate change and pollution has been completed to date. We will provide a description of the research area and identify gaps for future investigation, as well as lay the basis for future comparative work in IFS research on a global scale. We anticipate that this scoping review will provide knowledge about IFS changes and experiences related to climate change and pollution, actions taken to protect IFS, and characteristics of IFS. This focus on the impacts of environmental changes on IFS and responses to these changes is important in understanding community-driven solutions, which we hope will benefit Indigenous public health and provide knowledge for future research with Indigenous Peoples for IFS.

Through this review, we will help identify current demographic and methodological priorities and clarify what kinds of questions, methodologies, and conclusions are proliferating in the research area. Peer-reviewed academic literature is generally privileged in funding and dissemination, and therefore it is crucial to understand what messages are being sent about IFS through academic publications [16,17]. This is particularly important work as Indigenous voices have traditionally been excluded from institutional knowledge production [18].

In addition to the value of this review, our protocol is robust and limits bias by including various subject-specific databases, 2 independent reviewers, and consideration of region-specific terminology. However, this protocol is limited in that there are additional databases that could be included to expand the search. Our work also only includes primary research published in peer-reviewed journals; therefore, community projects that are published elsewhere are not included and may limit findings. While we do our best to use appropriate and context-specific terminology in our searches, it is possible that outdated terminology is included through database indexing and previous publications, and work using other terminology is missed.

Dissemination of this work will be completed through both academic publications and community conversations to ensure that communities can benefit from this knowledge. This review is important in understanding the primary research completed to date and areas for further exploration in the future. Ultimately, completing this review benefits not only academics but also Indigenous Peoples seeking to revitalize IFS for the health of their communities and future generations and will help to inform global climate policy directions.

## Appendix

Multimedia Appendix 1 Scoping review search string documentation.

## Abbreviations

IFS: Indigenous food systems
PRISMA: Preferred Reporting Items for Systematic Reviews and Meta-Analyses
PRISMA-ScR: Preferred Reporting Items for Systematic Reviews and Meta-Analyses Extension for Scoping Reviews

## Data Availability

Our search strategy is available in Multimedia Appendix 1. Journal articles at each of the review stages and review results will be published and available upon request.
