# Supplementary material for: Indigenous Food Systems Changes and Resiliency: Protocol for a Scoping Review
Source: JMIR Res Protoc. 2023 Apr 21;12:e41627. doi: 10.2196/41627 (PMC10163398; doi:10.2196/41627)
Supplement: Multimedia Appendix 1 [file resprot_v12i1e41627_app1.docx]

# Supplementary File: Search string documentation

1. MEDLINE (Ovid)
2. SCOPUS
3. International Bibliography of the Social Sciences (ProQuest)
4. Sociological Abstracts (Not including Social Services) (ProQuest)
5. Bibliography of Native North Americans (EBSCOHost)

All searches were performed June 1st 2021 and index terms were included for each database where index terms were available.

**1) MEDLINE**

(Ovid MEDLINE: Epub Ahead of Print, In-Process & Other Non-Indexed Citations, Ovid MEDLINE® Daily and Ovid MEDLINE® 1946-Present)

|  | **SEARCH TERM** | **RESULTS** |
| --- | --- | --- |
| **1** | exp Indigenous Peoples/ | 4787 |
| **2** | exp American Native Continental Ancestry Group/ | 21848 |
| **3** | exp Indians, Central American/ | 543 |
| **4** | exp Indians, North American/ | 17562 |
| **5** | exp Indians, South American/ | 3655 |
| **6** | exp Alaskan Natives/ | 4365 |
| **7** | exp Indigenous Canadians/ | 3954 |
| **8** | exp Inuits/ | 3939 |
| **9** | exp American Natives/ | 90 |
| **10** | exp Oceanic Ancestry Group/ | 10702 |
| **11** | 1 or 2 or 3 or 4 or 5 or 6 or 7 or 8 or 9 or 10 | 32594 |
| **12** | indigen*.mp | 44810 |
| **13** | first nation*.mp | 5020 |
| **14** | metis.mp | 391 |
| **15** | inuit*.mp | 4721 |
| **16** | aborigin*.mp | 10342 |
| **17** | maori*.mp | 3649 |
| **18** | (sami OR samis OR saami OR saamis).mp | 572 |
| **19** | 12 or 13 or 14 or 15 or 16 or 17 or 18 | 61265 |
| **20** | 11 or 19 | 78483 |
| **21** | (food ADJ2 system*1).mp | 4737 |
| **22** | (food ADJ5 sovereign*4).mp | 78 |
| **23** | (indigenous food*).mp | 135 |
| **24** | (tradition* ADJ1 food*).mp | 1577 |
| **25** | country food*.mp | 103 |
| **26** | food environment*.mp | 2948 |
| **27** | wild food*.mp | 215 |
| **28** | (food security or food insecurity).hw,kw | 2630 |
| **29** | 21 or 22 or 23 or 24 or 25 or 26 or 27 or 28 | 11808 |
| **30** | 20 and 29 | 774 |
| **31** | limit 30 to (english language and yr=“2016-current”) | 391 |

**2) SCOPUS**

|  | **SEARCH TERM** | **RESULTS** |
| --- | --- | --- |
| **1** | Indigen* | 149,296 |
| **2** | Indigen* People* | 37,632 |
| **3** | Aborigin* | 27,371 |
| **4** | (“Native American” OR ”American Native*”) | 14,807 |
| **5** | (“American Indian*” OR “Indians, North American” OR “North American Indian*”) | 26,980 |
| **6** | (“Central American Indian*” OR “Indians, Central American”) | 502 |
| **7** | (“South American Indian*” OR “Indians, South American”) | 3458 |
| **8** | “Alaska* Native*” | 4942 |
| **9** | (“Inuit*”) | 5374 |
| **10** | (“First Nation*”) | 10,400 |
| **11** | Metis | 1922 |
| **12** | (“Oceanic Ancestry Group”) | 8278 |
| **13** | Maori* | 8139 |
| **14** | (“Native Hawaii*” OR “Hawaii* Native*”) | 2113 |
| **15** | (“Indigen* Australia*” OR “Australia* Aborigin*”) | 7178 |
| **16** | “Torres Strait” | 3881 |
| **17** | (sami) OR (samis) OR (saami) OR (saamis) | 2737 |
| **18** | (“food system*” OR “system* of food”) | 12,516 |
| **19** | (food AND sovereignty) | 1483 |
| **20** | (“indigenous food*”) | 445 |
| **21** | (“tradition* food*” OR “food tradition*”) | 4372 |
| **22** | (“country food*”) | 769 |
| **23** | (food environment*”) | 4205 |
| **24** | (wild food*”) | 776 |
| **25** | (“food security” OR “food insecurity”) | 44,006 |
| **26** | ENGLISH: ( ( TITLE-ABS-KEY ( sami OR samis OR saami OR saamis ) ) OR ( TITLE-ABS-KEY ( "Torres Strait" ) ) OR ( TITLE-ABS-KEY ( "indigen* Australi*" OR "Australia* Aborigin*" ) ) OR ( TITLE-ABS-KEY ( "Native Hawaii*" OR "Hawaii* Native*" ) ) OR ( TITLE-ABS-KEY ( maori* ) ) OR ( TITLE-ABS-KEY ( "Oceanic Ancestry Group" ) ) OR ( TITLE-ABS-KEY ( metis ) ) OR ( TITLE-ABS-KEY ( "First Nation*" ) ) OR ( TITLE-ABS-KEY ( "Inuit*" ) ) OR ( TITLE-ABS-KEY ( "Alaska* Native*" ) ) OR ( TITLE-ABS-KEY ( "South American Indian*" OR "Indians, South American" ) ) OR ( TITLE-ABS-KEY ( "Central American Indian*" OR "Indians, Central American" ) ) OR ( TITLE-ABS-KEY ( "American Indian*" OR "Indians, North American" OR "North American Indian*" ) ) OR ( TITLE-ABS-KEY ( "Native American" OR "American Native*" ) ) OR ( TITLE-ABS-KEY ( aborigin* ) ) OR ( TITLE-ABS-KEY ( indigen* AND people* ) ) OR ( TITLE-ABS-KEY ( indigen* ) ) ) AND ( ( TITLE-ABS-KEY ( "food system*" OR "system* of food" ) ) OR ( TITLE-ABS-KEY ( food AND sovereignty ) ) OR ( TITLE-ABS-KEY ( "indigen* food*" ) ) OR ( TITLE-ABS-KEY ( "tradition* food*" OR "food tradition*" ) ) OR ( TITLE-ABS-KEY ( "country food*" ) ) OR ( TITLE-ABS-KEY ( "food environment*" ) ) OR ( TITLE-ABS-KEY ( "wild food*" ) ) OR ( TITLE-ABS-KEY ( "food security" OR "food insecurity" ) ) ) AND ( LIMIT-TO ( LANGUAGE , "English" ) ) | 2877 |
| **27** | 2016-PRESENT: ( ( TITLE-ABS-KEY ( sami OR samis OR saami OR saamis ) ) OR ( TITLE-ABS-KEY ( "Torres Strait" ) ) OR ( TITLE-ABS-KEY ( "indigen* Australi*" OR "Australia* Aborigin*" ) ) OR ( TITLE-ABS-KEY ( "Native Hawaii*" OR "Hawaii* Native*" ) ) OR ( TITLE-ABS-KEY ( maori* ) ) OR ( TITLE-ABS-KEY ( "Oceanic Ancestry Group" ) ) OR ( TITLE-ABS-KEY ( metis ) ) OR ( TITLE-ABS-KEY ( "First Nation*" ) ) OR ( TITLE-ABS-KEY ( "Inuit*" ) ) OR ( TITLE-ABS-KEY ( "Alaska* Native*" ) ) OR ( TITLE-ABS-KEY ( "South American Indian*" OR "Indians, South American" ) ) OR ( TITLE-ABS-KEY ( "Central American Indian*" OR "Indians, Central American" ) ) OR ( TITLE-ABS-KEY ( "American Indian*" OR "Indians, North American" OR "North American Indian*" ) ) OR ( TITLE-ABS-KEY ( "Native American" OR "American Native*" ) ) OR ( TITLE-ABS-KEY ( aborigin* ) ) OR ( TITLE-ABS-KEY ( indigen* AND people* ) ) OR ( TITLE-ABS-KEY ( indigen* ) ) ) AND ( ( TITLE-ABS-KEY ( "food system*" OR "system* of food" ) ) OR ( TITLE-ABS-KEY ( food AND sovereignty ) ) OR ( TITLE-ABS-KEY ( "indigen* food*" ) ) OR ( TITLE-ABS-KEY ( "tradition* food*" OR "food tradition*" ) ) OR ( TITLE-ABS-KEY ( "country food*" ) ) OR ( TITLE-ABS-KEY ( "food environment*" ) ) OR ( TITLE-ABS-KEY ( "wild food*" ) ) OR ( TITLE-ABS-KEY ( "food security" OR "food insecurity" ) ) ) AND ( LIMIT-TO ( LANGUAGE , "English" ) ) AND ( LIMIT-TO ( PUBYEAR , 2021 ) OR LIMIT-TO ( PUBYEAR , 2020 ) OR LIMIT-TO ( PUBYEAR , 2019 ) OR LIMIT-TO ( PUBYEAR , 2018 ) OR LIMIT-TO ( PUBYEAR , 2017 ) OR LIMIT-TO ( PUBYEAR , 2016 ) ) | 1444 |

**3) International Bibliography of the Social Sciences (ProQuest)**

|  | **SEARCH TERM** | **RESULTS** |
| --- | --- | --- |
| **1** | ti,ab,su(“Indigen*”) | 50,565 |
| **2** | ti,ab,su(“Indigen* People*”) | 12,066 |
| **3** | MAINSUBJECT.EXACT("Native peoples") | 10,295 |
| **4** | ti,ab,su(“Aboriginal”) | 7651 |
| **5** | ti,ab,su(“Native American” OR ”American Native*”) | 3142 |
| **6** | MAINSUBJECT.EXACT("Native North Americans") | 7116 |
| **7** | ti,ab,su("american indian" OR "american indians" OR “Indians, North American” OR “North American Indian*”) | 7111 |
| **8** | ti,ab,su(“Central American Indian*” OR “Indians, Central American”) | 17 |
| **9** | ti,ab,su(“South American Indian*” OR “Indians, South American”) | 154 |
| **10** | ti,ab,su(“Alaska* Native*”) | 3136 |
| **11** | ti,ab,su(“Inuit*”) | 3195 |
| **12** | MAINSUBJECT.EXACT("Inuit") | 2548 |
| **13** | ti,ab,su("first nation" OR "first nations") | 1687 |
| **14** | ti,ab,su("Metis") | 669 |
| **15** | MAINSUBJECT.EXACT("Metis") | 144 |
| **16** | ti,ab,su(“Oceanic Ancestry Group” OR "pacific islander" OR "pacific islanders”) | 719 |
| **17** | MAINSUBJECT.EXACT("Pacific Islander people") | 170 |
| **18** | ti,ab,su(“maori" OR "maoris") | 2667 |
| **19** | ti,ab,su("native hawaiian" OR "native hawaiians") | 247 |
| **20** | ti,ab,su(“Indigen* Australia*” OR “Australia* Aborigin*”) | 1625 |
| **21** | ti,ab,su(“Torres Strait”) | 628 |
| **22** | ti,ab,su("sami" OR "samis" OR "saami" OR "saamis") | 894 |
| **23** | ti,ab,su(“Indigen*”) OR ti,ab,su(“Indigen* People*”) OR MAINSUBJECT.EXACT("Native peoples") OR ti,ab,su(“Aboriginal”) OR ti,ab,su(“Native American” OR ”American Native*”) OR MAINSUBJECT.EXACT("Native North Americans") OR ti,ab,su("american indian" OR "american indians" OR “Indians, North American” OR “North American Indian*”) OR ti,ab,su(“Central American Indian*” OR “Indians, Central American”) OR ti,ab,su(“South American Indian*” OR “Indians, South American”) OR ti,ab,su(“Alaska* Native*”) OR ti,ab,su(“Inuit*”) OR MAINSUBJECT.EXACT("Inuit") OR ti,ab,su("first nation" OR "first nations") OR ti,ab,su("Metis") OR MAINSUBJECT.EXACT("Metis") OR ti,ab,su(“Oceanic Ancestry Group” OR "pacific islander" OR "pacific islanders”) OR MAINSUBJECT.EXACT("Pacific Islander people") OR ti,ab,su(“maori" OR "maoris") OR ti,ab,su("native hawaiian" OR "native hawaiians") OR ti,ab,su(“Torres Strait”) OR ti,ab,su("sami" OR "samis" OR "saami" OR "saamis") | 74,098 |
| **24** | ti,ab,su("food system" OR "food systems") | 1724 |
| **25** | MAINSUBJECT.EXACT(“Food") | 15,560 |
| **26** | MAINSUBJECT.EXACT("Food supply”) | 4174 |
| **27** | ti,ab,su("Food Sovereignty") | 482 |
| **28** | ti,ab,su("indigenous food" OR "indigenous foods") | 54 |
| **29** | MAINSUBJECT.EXACT(“Hunter-gatherers") | 1416 |
| **30** | ti,ab,su(“traditional food” OR “traditional foods” OR "food tradition" OR "food traditions") | 290 |
| **31** | MAINSUBJECT.EXACT("Folk medicine") | 441 |
| **32** | ti,ab,su("country food" OR "country foods") | 49 |
| **33** | ti,ab,su("food environment” OR "food environments") | 242 |
| **34** | ti,ab,su("wild food" OR "wild foods") | 80 |
| **35** | MAINSUBJECT.EXACT("Foraging behavior") | 276 |
| **36** | ti,ab,su(“food security” OR “food insecurity”) | 7310 |
| **37** | MAINSUBJECT.EXACT("Food security") | 4775 |
| **38** | ti,ab,su("food system" OR "food systems") OR MAINSUBJECT.EXACT(“Food") OR MAINSUBJECT.EXACT("Food supply”) OR ti,ab,su("Food Sovereignty") OR ti,ab,su("indigenous food" OR "indigenous foods") OR MAINSUBJECT.EXACT(“Hunter-gatherers") OR ti,ab,su(“traditional food” OR “traditional foods” OR "food tradition" OR "food traditions") OR MAINSUBJECT.EXACT("Folk medicine") OR ti,ab,su("country food" OR "country foods") OR ti,ab,su("food environment” OR "food environments") OR ti,ab,su("wild food" OR "wild foods") OR MAINSUBJECT.EXACT("Foraging behavior") OR ti,ab,su(“food security” OR “food insecurity”) OR MAINSUBJECT.EXACT("Food security") | 1,391,087 |
| **39** | (ti,ab,su(“Indigen*”) OR ti,ab,su(“Indigen* People*”) OR MAINSUBJECT.EXACT("Native peoples") OR ti,ab,su(“Aboriginal”) OR ti,ab,su(“Native American” OR ”American Native*”) OR MAINSUBJECT.EXACT("Native North Americans") OR ti,ab,su("american indian" OR "american indians" OR “Indians, North American” OR “North American Indian*”) OR ti,ab,su(“Central American Indian*” OR “Indians, Central American”) OR ti,ab,su(“South American Indian*” OR “Indians, South American”) OR ti,ab,su(“Alaska* Native*”) OR ti,ab,su(“Inuit*”) OR MAINSUBJECT.EXACT("Inuit") OR ti,ab,su("first nation" OR "first nations") OR ti,ab,su("Metis") OR MAINSUBJECT.EXACT("Metis") OR ti,ab,su(“Oceanic Ancestry Group” OR "pacific islander" OR "pacific islanders”) OR MAINSUBJECT.EXACT("Pacific Islander people") OR ti,ab,su(“maori" OR "maoris") OR ti,ab,su("native hawaiian" OR "native hawaiians") OR ti,ab,su(“Torres Strait”) OR ti,ab,su("sami" OR "samis" OR "saami" OR "saamis")) AND (ti,ab,su("food system" OR "food systems") OR MAINSUBJECT.EXACT(“Food") OR MAINSUBJECT.EXACT("Food supply”) OR ti,ab,su("Food Sovereignty") OR ti,ab,su("indigenous food" OR "indigenous foods") OR MAINSUBJECT.EXACT(“Hunter-gatherers") OR ti,ab,su(“traditional food” OR “traditional foods” OR "food tradition" OR "food traditions") OR MAINSUBJECT.EXACT("Folk medicine") OR ti,ab,su("country food" OR "country foods") OR ti,ab,su("food environment” OR "food environments") OR ti,ab,su("wild food" OR "wild foods") OR MAINSUBJECT.EXACT("Foraging behavior") OR ti,ab,su(“food security” OR “food insecurity”) OR MAINSUBJECT.EXACT("Food security")) | 13,062 |
| **40** | LA(English) | 4,663,417 |
| **41** | YR(2016-2021) | 621,123 |
| **42** | SCHOL(yes) | 4,892,182 |
| **43** | PEER(yes) | 3,822,988 |
| **44** | LA(English) AND YR(2016-2021) AND SCHOL(yes) AND PEER(yes) | 494,137 |
| **45** | 39 AND 44 | 275 |

**4) Sociological Abstracts (Not including Social Services) (ProQuest)**

|  | **SEARCH TERM** | **RESULTS** |
| --- | --- | --- |
| **1** | ti,ab,su(“Indigen*”) | 25,110 |
| **2** | ti,ab,su(“Indigen* People*”) | 8203 |
| **3** | MAINSUBJECT.EXACT.EXPLODE("Indigenous Populations") | 8207 |
| **4** | ti,ab,su(“Aboriginal”) | 5273 |
| **5** | MAINSUBJECT.EXACT("Traditional Societies") | 3803 |
| **6** | MAINSUBJECT.EXACT("Hunting and Gathering Societies") | 294 |
| **7** | MAINSUBJECT.EXACT("Nomadic Societies") | 404 |
| **8** | MAINSUBJECT.EXACT("Decolonization") | 5901 |
| **9** | MAINSUBJECT.EXACT("Colonization") | 1462 |
| **10** | ti,ab,su(“Native American” OR ”American Native*”) | 3225 |
| **11** | MAINSUBJECT.EXACT("American Indian Reservations") | 224 |
| **12** | ti,ab,su("american indian" OR "american indians" OR “Indians, North American” OR “North American Indian*”) | 7685 |
| **13** | MAINSUBJECT.EXACT("American Indians") | 4930 |
| **14** | MAINSUBJECT.EXACT("Mayans") | 350 |
| **15** | ti,ab,su(“Alaska* Native*”) | 1746 |
| **16** | ti,ab,su(“Inuit*”) | 886 |
| **17** | MAINSUBJECT.EXACT("Eskimos") | 433 |
| **18** | ti,ab,su("first nation" OR "first nations") | 1039 |
| **19** | ti,ab,su("Metis") | 402 |
| **20** | ti,ab,su(“Oceanic Ancestry Group” OR "pacific islander" OR "pacific islanders”) | 632 |
| **21** | MAINSUBJECT.EXACT("Oceanic Cultural Groups") | 327 |
| **22** | ti,ab,su(“maori" OR "maoris") | 1231 |
| **23** | ti,ab,su("native hawaiian" OR "native hawaiians") | 282 |
| **24** | ti,ab,su(“Indigen* Australia*” OR “Australia* Aborigin*”) | 1041 |
| **25** | MAINSUBJECT.EXACT("Aboriginal Australians") | 1931 |
| **26** | ti,ab,su(“Torres Strait”) | 237 |
| **27** | ti,ab,su("sami" OR "samis" OR "saami" OR "saamis") | 345 |
| **28** | ti,ab,su(“Indigen*”) OR ti,ab,su(“Indigen* People*”) OR MAINSUBJECT.EXACT.EXPLODE("Indigenous Populations") OR ti,ab,su(“Aboriginal”) OR MAINSUBJECT.EXACT("Traditional Societies") OR MAINSUBJECT.EXACT("Hunting and Gathering Societies") OR MAINSUBJECT.EXACT("Nomadic Societies") OR MAINSUBJECT.EXACT("Decolonization") OR MAINSUBJECT.EXACT("Colonization") OR ti,ab,su(“Native American” OR ”American Native*”) OR MAINSUBJECT.EXACT("American Indian Reservations") OR ti,ab,su("american indian" OR "american indians" OR “Indians, North American” OR “North American Indian*”) OR MAINSUBJECT.EXACT("American Indians") OR MAINSUBJECT.EXACT("Mayans") OR ti,ab,su(“Alaska* Native*”) OR ti,ab,su(“Inuit*”) OR MAINSUBJECT.EXACT("Eskimos") OR ti,ab,su("first nation" OR "first nations") OR ti,ab,su("Metis") OR ti,ab,su(“Oceanic Ancestry Group” OR "pacific islander" OR "pacific islanders”) OR MAINSUBJECT.EXACT("Oceanic Cultural Groups") OR ti,ab,su(“maori" OR "maoris") OR ti,ab,su("native hawaiian" OR "native hawaiians") OR ti,ab,su(“Indigen* Australia*” OR “Australia* Aborigin*”) OR MAINSUBJECT.EXACT("Aboriginal Australians") OR ti,ab,su(“Torres Strait”) OR ti,ab,su("sami" OR "samis" OR "saami" OR "saamis") | 46,802 |
| **29** | ti,ab,su("food system" OR "food systems") | 1300 |
| **30** | MAINSUBJECT.EXACT.EXPLODE("Food") | 5540 |
| **31** | MAINSUBJECT.EXACT("Traditional Medicine") | 1147 |
| **32** | MAINSUBJECT.EXACT("Hunting") | 767 |
| **33** | ti,ab,su("Food Sovereignty") | 435 |
| **34** | ti,ab,su("indigenous food" OR "indigenous foods") | 35 |
| **35** | ti,ab,su(“traditional food” OR “traditional foods” OR "food tradition" OR "food traditions") | 166 |
| **36** | ti,ab,su("country food" OR "country foods") | 14 |
| **37** | ti,ab,su("food environment” OR "food environments") | 147 |
| **38** | ti,ab,su("wild food" OR "wild foods") | 43 |
| **39** | ti,ab,su(“food security” OR “food insecurity”) | 2580 |
| **40** | MAINSUBJECT.EXACT("Food Security") | 1575 |
| **41** | ti,ab,su("food system" OR "food systems") OR MAINSUBJECT.EXACT.EXPLODE("Food") OR MAINSUBJECT.EXACT("Traditional Medicine") OR MAINSUBJECT.EXACT("Hunting") OR ti,ab,su("Food Sovereignty") OR ti,ab,su("indigenous food" OR "indigenous foods") OR ti,ab,su(“traditional food” OR “traditional foods” OR "food tradition" OR "food traditions") OR ti,ab,su("country food" OR "country foods") OR ti,ab,su("food environment” OR "food environments") OR ti,ab,su("wild food" OR "wild foods") OR ti,ab,su(“food security” OR “food insecurity”) OR MAINSUBJECT.EXACT("Food Security") | 10,330 |
| **42** | 28 AND 41 | 961 |
| **43** | LA(English) | 1,658,329 |
| **44** | YR(2016-2021) | 218,309 |
| **45** | SCHOL(yes) | 1,781,708 |
| **46** | PEER(yes) | 1,510,775 |
| **47** | LA(English) AND YR(2016-2021) AND SCHOL(yes) AND PEER(yes) | 173,930 |
| **48** | ((ti,ab,su(“Indigen*”) OR ti,ab,su(“Indigen* People*”) OR MAINSUBJECT.EXACT.EXPLODE("Indigenous Populations") OR ti,ab,su(“Aboriginal”) OR MAINSUBJECT.EXACT("Traditional Societies") OR MAINSUBJECT.EXACT("Hunting and Gathering Societies") OR MAINSUBJECT.EXACT("Nomadic Societies") OR MAINSUBJECT.EXACT("Decolonization") OR MAINSUBJECT.EXACT("Colonization") OR ti,ab,su(“Native American” OR ”American Native*”) OR MAINSUBJECT.EXACT("American Indian Reservations") OR ti,ab,su("american indian" OR "american indians" OR “Indians, North American” OR “North American Indian*”) OR MAINSUBJECT.EXACT("American Indians") OR MAINSUBJECT.EXACT("Mayans") OR ti,ab,su(“Alaska* Native*”) OR ti,ab,su(“Inuit*”) OR MAINSUBJECT.EXACT("Eskimos") OR ti,ab,su("first nation" OR "first nations") OR ti,ab,su("Metis") OR ti,ab,su(“Oceanic Ancestry Group” OR "pacific islander" OR "pacific islanders”) OR MAINSUBJECT.EXACT("Oceanic Cultural Groups") OR ti,ab,su(“maori" OR "maoris") OR ti,ab,su("native hawaiian" OR "native hawaiians") OR ti,ab,su(“Indigen* Australia*” OR “Australia* Aborigin*”) OR MAINSUBJECT.EXACT("Aboriginal Australians") OR ti,ab,su(“Torres Strait”) OR ti,ab,su("sami" OR "samis" OR "saami" OR "saamis")) AND (ti,ab,su("food system" OR "food systems") OR MAINSUBJECT.EXACT.EXPLODE("Food") OR MAINSUBJECT.EXACT("Traditional Medicine") OR MAINSUBJECT.EXACT("Hunting") OR ti,ab,su("Food Sovereignty") OR ti,ab,su("indigenous food" OR "indigenous foods") OR ti,ab,su(“traditional food” OR “traditional foods” OR "food tradition" OR "food traditions") OR ti,ab,su("country food" OR "country foods") OR ti,ab,su("food environment” OR "food environments") OR ti,ab,su("wild food" OR "wild foods") OR ti,ab,su(“food security” OR “food insecurity”) OR MAINSUBJECT.EXACT("Food Security"))) AND (LA(English) AND YR(2016-2021) AND SCHOL(yes) AND PEER(yes)) | 169 |

**5) Bibliography of Native North Americans (EBSCOHost)**

|  | **SEARCH TERM** | **RESULTS** |
| --- | --- | --- |
| **1** | TI(Indigen*) OR AB(Indigen*) OR SU(Indigen*) OR KW(Indigen*) | 18,720 |
| **2** | TI(Indigen* People*) OR AB(Indigen* People*) OR SU(Indigen* People*) OR KW(Indigen* People*) | 12,386 |
| **3** | TI(Aboriginal) OR AB(Aboriginal) OR SU(Aboriginal) OR KW(Aboriginal) | 10,203 |
| **4** | ( TI(Native American” OR American Native*) ) OR ( AB(Native American OR American Native*) ) OR ( SU(Native American OR American Native*) ) OR ( KW(Native American OR American Native*) ) | 38,946 |
| **5** | ( TI(american indian OR american indians OR Indians, North American OR North American Indian*) ) OR ( AB(american indian OR american indians OR Indians, North American OR North American Indian*) ) OR ( SU(american indian OR american indians OR Indians, North American OR North American Indian*) ) OR ( KW(american indian OR american indians OR Indians, North American OR North American Indian*) ) | 15,884 |
| **6** | ( TI(Central American Indian* OR Indians, Central American) ) OR ( AB(Central American Indian* OR Indians, Central American) ) OR ( SU(Central American Indian* OR Indians, Central American) ) OR ( KW(Central American Indian* OR Indians, Central American) ) | 19 |
| **7** | ( TI(South American Indian* OR Indians, South American) ) OR ( AB(South American Indian* OR Indians, South American) ) OR ( SU(South American Indian* OR Indians, South American) ) OR ( KW(South American Indian* OR Indians, South American) ) | 94 |
| **8** | TI(Alaska* Native*) OR AB(Alaska* Native*) OR SU(Alaska* Native*) OR KW(Alaska* Native*) | 2898 |
| **9** | TI (Inuit *) OR AB (Inuit *) OR SU (Inuit *) OR KW (Inuit *) | 7688 |
| **10** | ( TI(first nation OR first nations) ) OR ( AB(first nation OR first nations) ) OR ( SU(first nation OR first nations) ) OR ( KW(first nation OR first nations) ) | 10,339 |
| **11** | TI(Metis) OR AB(Metis) OR SU(Metis) OR KW(Metis) | 2337 |
| **12** | ( TI(Oceanic Ancestry Group OR pacific islander OR pacific islanders) ) OR ( AB(Oceanic Ancestry Group OR pacific islander OR pacific islanders) ) OR ( SU(Oceanic Ancestry Group OR pacific islander OR pacific islanders) ) OR ( KW(Oceanic Ancestry Group OR pacific islander OR pacific islanders) ) | 59 |
| **13** | ( TI(maori OR maoris) ) OR ( AB(maori OR maoris) ) OR ( SU(maori OR maoris) ) OR ( KW(maori OR maoris) ) | 191 |
| **14** | ( TI(native hawaiian OR native hawaiians) ) OR ( AB(native hawaiian OR native hawaiians) ) OR ( SU(native hawaiian OR native hawaiians) ) OR ( KW(native hawaiian OR native hawaiians) ) | 261 |
| **15** | ( TI(Indigen* Australia* OR Australia* Aborigin*) ) OR ( AB(Indigen* Australia* OR Australia* Aborigin*) ) OR ( SU(Indigen* Australia* OR Australia* Aborigin*) ) OR ( KW(Indigen* Australia* OR Australia* Aborigin*) ) | 241 |
| **16** | TI(Torres Strait) OR AB(Torres Strait) OR SU(Torres Strait) OR KW(Torres Strait) | 63 |
| **17** | ( TI(sami OR samis OR saami OR saamis) ) OR ( AB(sami OR samis OR saami OR saamis) ) OR ( SU(sami OR samis OR saami OR saamis) ) OR ( KW(sami OR samis OR saami OR saamis) ) | 96 |
| **18** | DE “metis" | 565 |
| **19** | DE “aboriginal canadians” | 1223 |
| **20** | DE “native americans” | 9720 |
| **21** | DE “maori (new zealand people)” | 82 |
| **22** | DE “first nations of canada” | 2457 |
| **23** | DE “hawaiians" | 130 |
| **24** | DE "sami (european people)" | 43 |
| **25** | DE “indigenous peoples” | 5283 |
| **26** | DE “pacific islander americans” | 2 |
| **27** | DE "pacific islanders” | 8 |
| **28** | DE “alaska natives” | 1313 |
| **29** | DE “inuit" | 704 |
| **30** | DE “mayas” | 33 |
| **31** | DE "indigenous australians” | 57 |
| **32** | DE "torres strait islanders” | 14 |
| **33** | S1 OR S2 OR S3 OR S4 OR S5 OR S6 OR S7 OR S8 OR S9 OR S10 OR S11 OR S12 OR S13 OR S14 OR S15 OR S16 OR S17 OR S18 OR S19 OR S20 OR S21 OR S22 OR S23 OR S24 OR S25 OR S26 OR S27 OR S28 OR S29 OR S30 OR S31 OR S32)) AND (S1 OR S2 OR S3 OR S4 OR S5 OR S6 OR S7 OR S8 OR S9 OR S10 OR S11 OR S12 OR S13 OR S14 OR S15 OR S16 OR S17 OR S18 OR S19 OR S20 OR S21 OR S22 OR S23 OR S24 OR S25 OR S26 OR S27 OR S28 OR S29 OR S30 OR S31 OR S32 | 78,645 |
| **34** | ( TI(food system OR food systems) ) OR ( AB(food system OR food systems) ) OR ( SU(food system OR food systems) ) OR ( KW(food system OR food systems) ) | 136 |
| **35** | TI(Food Sovereignty) OR AB(Food Sovereignty) OR SU(Food Sovereignty) OR KW(Food Sovereignty) | 127 |
| **36** | ( TI(indigenous food OR indigenous foods) ) OR ( AB(indigenous food OR indigenous foods) ) OR ( SU(indigenous food OR indigenous foods) ) OR ( KW(indigenous food OR indigenous foods) ) | 81 |
| **37** | ( TI(traditional food OR traditional foods OR food tradition OR food traditions) ) OR ( AB(traditional food OR traditional foods OR food tradition OR food traditions) ) OR ( SU(traditional food OR traditional foods OR food tradition OR food traditions) ) OR ( KW(traditional food OR traditional foods OR food tradition OR food traditions) ) | 219 |
| **38** | ( TI(country food OR country foods) ) OR ( AB(country food OR country foods) ) OR ( SU(country food OR country foods) ) OR ( KW(country food OR country foods) ) | 30 |
| **39** | ( TI(food environment OR food environments) ) OR ( AB(food environment OR food environments) ) OR ( SU(food environment OR food environments) ) OR ( KW(food environment OR food environments) ) | 108 |
| **40** | ( TI(wild food OR wild foods) ) OR ( AB(wild food OR wild foods) ) OR ( SU(wild food OR wild foods) ) OR ( KW(wild food OR wild foods) ) | 50 |
| **41** | ( TI(food security OR food insecurity) ) OR ( AB(food security OR food insecurity) ) OR ( SU(food security OR food insecurity) ) OR ( KW(food security OR food insecurity) ) | 305 |
| **42** | DE “Food" | 335 |
| **43** | DE “Food supply” | 98 |
| **44** | DE “Hunter-gatherer societies” | 202 |
| **45** | DE “natural foods” | 82 |
| **46** | DE “hunting” | 879 |
| **47** | DE "Food security" | 240 |
| **48** | DE "Food sovereignty” | 97 |
| **49** | TI(food) OR AB(food) OR SU(food) OR KW(food) | 5971 |
| **50** | S34 OR S35 OR S36 OR S37 OR S38 OR S39 OR S40 OR S41 OR S42 OR S43 OR S44 OR S45 OR S46 OR S47 OR S48 OR S49 | 6990 |
| **51** | S33 AND S50 | 1502 |
| **52** | Limiters to add to line 51 (use check boxes) : 2016-2021, “Academic Journal” | 101 |
